# Anemia in patients ten years after bariatric surgery

**DOI:** 10.1038/s41366-024-01675-4

**Published:** 2024-11-09

**Authors:** Mimmi Karlsson, Johan Ottosson, Spencer Clarkson, Klas Sjöberg

**Affiliations:** 1 Department of Gastroenterology and Nutrition, Skåne University Hospital, Malmö, Sweden; 2 Department of Surgery, Faculty of Medicine and Health, Örebro University, Örebro, Sweden; 3 Digitalisation MT and IT, Medicon Village, Lund, Sweden; 4 Department of Clinical Sciences, Malmö, Lund University, Malmö, Sweden

**Keywords:** Digestive signs and symptoms, Feeding behaviour

## Abstract

**Background:**

More than 10% of the global population has a BMI above 35. Bariatric surgery is an efficient way to treat this condition. Unfortunately, there is a risk of nutritional deficiencies. The number of studies after a longer time span is scarce. The aim of this study was to determine the occurrence of anaemia five and ten years after bariatric surgery and how it was related to substitution therapy.

**Patients and methods:**

Registry data from individuals having primary bariatric surgery in the Scandinavian Obesity Surgery Registry (SOReg) from 2007 to 2022 and with a follow-up at five or ten years was retrieved. Demographic data including weight, as well as method of surgery, Hb levels, supplementation, PPI use and stomal ulcerations were recorded.

**Results:**

In total, 39,992 individuals (mean age 41 years, range 18–74, 77% women) could be included. The majority, 78%, had undergone laparoscopic Roux-en-Y gastric bypass. After five years, 2838/13,944 women (20.3%) and 456/4049 men (11.2%) had anaemia. After ten years, 644/3400 women (18.9%) and 178/947 men (18.8%) had anaemia. The use of oral iron increased from 40 to 45%, and the need for parenteral iron intake increased from 5 to 11%.

**Conclusions:**

Anaemia is a significant but manageable condition five and ten years after bariatric surgery. Despite the prescription of oral iron supplements to 45% ten years after surgery, the Hb levels could still not be fully restored. Consequently, the importance of follow-up visits and continuous supplementation is emphasised.

## Introduction

The prevalence of obesity is increasing worldwide. Between the years 1975 and 2014, the global age-standardised prevalence, defined as body mass index (BMI) ≥ 30 kg/m², has increased from 3.2% to 10.8% in men, and from 6.4% to 14.9% in women [1]. In 2014, 2.3% of the world’s men and 5.0% of the world’s women had a BMI ≥ 35 kg/m² [1]. Obesity is, in turn, a risk factor for a range of health issues e.g., metabolic syndrome, type 2 diabetes, hypertension, hyperlipidaemia, and cardiovascular disease, as well as a risk factor for cancer [2, 3] and a shorter life expectancy compared to the overall population [4].

Many trials and programs have been initiated with the aim to reduce BMI and, at the same time, reduce the health risks associated with obesity. Bariatric surgery has proven to be the most efficient treatment for reducing and then keeping a lower BMI over time [2]. On average, 5000 bariatric surgeries are carried out in Sweden each year. The positive outcomes after bariatric surgery are well documented with reduced cardiovascular disease, improvement or even remission of type 2 diabetes as well as protection against development of type 2 diabetes [2, 5]. The Swedish Obese Subjects (SOS) study, a prospective controlled trial, has, among other things, shown a decrease in overall mortality as well as a decrease in the risk of cancer after bariatric surgery in people with obesity [6, 7].

The Scandinavian Obesity Surgery Registry (SOReg) is a national research and quality register that started in 2007 covering virtually all bariatric surgical procedures in Sweden. The register is continuously validated, and registrations have been shown to have very high validity [8]. More than 80,000 operations have been registered, and follow-up visits are registered at 6 weeks, 1 year, 2 years, 5 years, and 10 years after the operation. At the follow-ups, the coverage rate varies between 40 and 95%.

The complications second to bariatric surgery vary with the surgical methods used. However, surgical complications, as well as dysmotility, may occur in all types of surgical procedures. These side effects include bowel obstruction, stomal ulcerations, and gallstone problems [9]. Several other adverse effects after bariatric surgery have been described. These include increased mental illness sometimes with suicidal tendencies or risk of alcohol dependence, and furthermore, deficiencies of various nutrients, that among others risk resulting in an increased incidence of anaemia [10, 11]. Patients going through bariatric surgery have an increased risk of anaemia already after a shorter period of follow-up. Furthermore, the anaemia resumes after standard substitution is given [12]. Regardless of the cause of anaemia the condition eventually leads to fatigue and weakness. Anaemia has also been associated with a lower quality of life (QoL) [13].

In patients that have undergone bariatric surgery the risk for anaemia in the long run, i.e., after five and ten years, has been scarcely investigated. In a larger group of 959 patients (where 85% were female) that underwent laparoscopic Roux-en-Y gastric bypass 51% had iron deficiency ten years later. The mean haemoglobin (Hb) levels decreased from 135 to 116 g/l [14]. In another investigation with the same time span 47% of 348 patients had iron deficiency with a Hb fall from 138 to 124 g/l after 36 months [15]. In a Norwegian study iron deficiency (based on ferritin levels) occurred in 44% of 530 patients after 10–15 years. The Hb levels were divided into subgroups making comparisons with other reports difficult [16]. In a major prospective “Swedish Obese Subjects” study comprising 4047 patients in 2007 underwent bariatric surgery (266 gastric bypass group, 1365 vertical-banded gastroplasty group, and 376 gastric banding) and 2040 received ordinary non-surgical obesity care. The risk for anaemia after a median of ten years was markedly increased in the groups that had been operated compared to the control group (HR 5.05, 2.67, and 2.76, respectively) [17]. Based on the published data this far it would be valuable to follow up on these results in a larger cohort with follow-up according to present guidelines.

The purpose of this study was to determine the occurrence of anaemia after bariatric surgery in a national cohort over a longer time span and how it was related to substitution therapy, and complications.

## Patients and methods

**Fig. 1:**
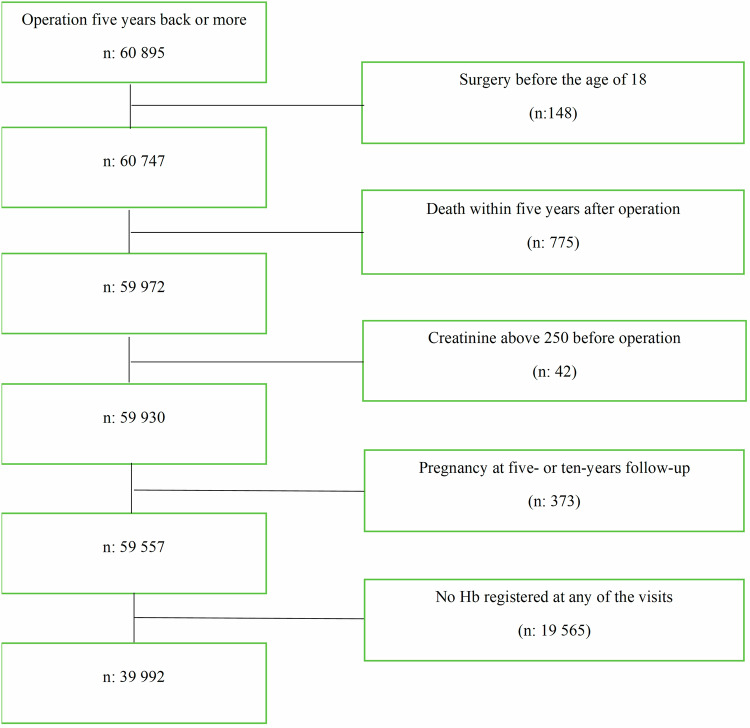
Selection process. The figure illustrates the procedure used to select patients that were relevant for the study.

Data has been retrieved from the Scandinavian Obesity Surgery Registry (SOReg) database collected until December 2022. All patients in the SOReg database who had undergone primary bariatric surgery (i.e., Roux-en-Y gastric bypass (GBY), Sleeve Gastrectomy (SG) or a Bilio-pancreatic diversion with duodenal switch (DS)) with a follow-up period of at least five years were included. In other words, only patients who had gone through bariatric surgery between January 2007 and December 2017 were included. In total 52 centres have contributed with data to this study. Patients with bariatric surgery before the age of 18, with a registered date of death before a five-year follow-up period, creatinine above 250, and pregnancy in close proximity to their five- or ten-year follow-up were excluded. Before exclusion the population consisted of 60,895 individuals. See Fig. 1.

Demographic data regarding sex, age at the time of surgery and surgical method was registered. At five- and ten-year follow-ups weight, BMI, smoking habits, mean Hb levels, percentage with anaemia and supplementary treatment were also retrieved. Treatment with proton pump inhibitors (PPI) and stomal ulcerations were also recorded since these factors can also affect Hb levels. Anaemia was defined according to the WHO definition in which the blood cell level (Hb) is under 120 g/L for women and under 130 g/L for men [18].

Since Hb was not analysed at all visits there was some missingness of data. However, we have used linear mixed model analyses to account for missing data by calculating estimates for them. We used IBM SPSS software version 28. Furthermore, a dropout analysis was carried out with an independent two-sided *t*-test. A *p*-value below 0.05 was considered significant.

The study was approved by the Swedish Ethical Review Authority (protocol number 2020-02233). The study is registered-based with anonymized data prior to analysis. Since the present study was strictly register-based informed consent was not required. Waiver of informed consent was approved by the Swedish Ethical Review Authority. All methods used in the study were carried out in accordance with relevant guidelines and regulations.

## Results

**Fig. 2:**
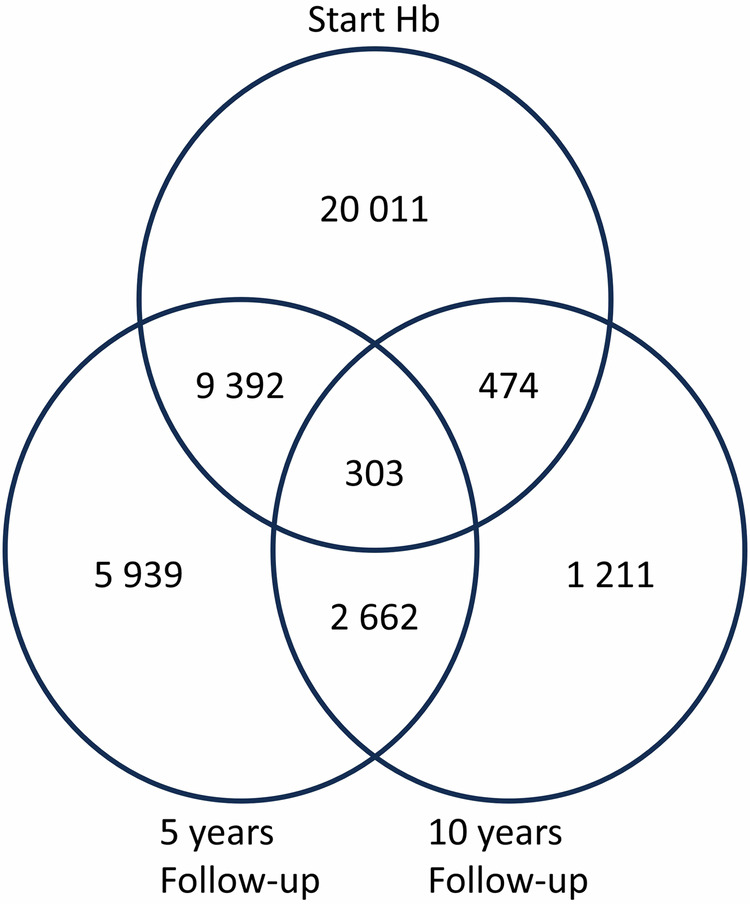
Number of patients with analysed Hb in the different groups. The upper circle identifies all that left Hb at start. The lower circle on the left-hand side identfies all that left Hb at 5 years follow-up. The lower circle at the right-hand side identifies all that left Hb at 10 years follow-up.

After the exclusion of confounding factors as described in Patients and Methods the study comprised 59,557 individuals collected from 52 centres ranging from 1 to 6788 patients, mean number from each centre of 1145 patients with a mean age of 41 at the time of surgery, range 18–74, 76% women. Since registration of the Hb levels was not possible in SOReg between 2007 and 2011 only 50% in the group prior to surgery had any Hb level stated. At five- and ten-years follow-up the percentages were 52% (range 0–79% from the different centres) and 11% (0–38% from the different centres), respectively. Consequently, 19,565 of these 59,557 patients did not have any Hb value registered at any of their visits and had to be excluded as well. In order to maximise the number of included patients, all that had left at least one Hb at any of the visits (before surgery and at five- and ten-years follow-up, respectively) were included. Consequently, the number of patients that finally could be included in the analysis of anaemia was 39,992 patients with a registered Hb value at one or more of the control visits, i.e., 29,877 patients prior to surgery, 17,993 after five years and 4347 after ten years. See Table 1 and Fig. 2. Since registration of the Hb levels was not carried out in all patients consecutively at all visits the cohorts from the start and at 5- and 10-years follow-ups do not consist of exactly the same patients. See Table 2. The Hb mean level was lower at the five-year follow-up compared to preoperative Hb (*p* < 0.001). Although the difference was lower between the Hb levels at the five-year follow-up compared to the ten-year follow-up, the difference was highly significant (*p* < 0.001). For the group that was followed for ten years (4347 patients) the mean Hb level was 131.6 g/l, a decrease compared to the level before surgery (140.1 g/l in 474 patients) but comparable to the levels at five years (130.4 g/l in 2662 patients). See Table 2.

**Table 1:** Characteristics of the study population prior to bariatric surgery, and at follow-up at five and ten years, respectively.

	Prior to surgery	(%)	Follow-up 5 years	(%)	Follow-up 10 years	(%)
**Total population**						
Number	29,877		17,993		4347	
**Sex**						
Women	22,943	(76.8)	13,944	(77.5)	3400	(78.2)
Men	6934	(23.2)	4049	(22.5)	947	(21.8)
**Age at surgery (years)**						
Mean	41.0		42.8		42.6	
(range)	(18–74)		(18–73)		(18–73)	
**Method of surgery**						
GBY*	23,453	(78.5)	15,737	(87.5)	4271	(98.3)
SG**	6248	(20.9)	2053	(11.4)	17	(0.3)
DS***	176	(0.6)	203	(1.1)	64	(1.5)
**Weight (kg)**						
Mean	118.1		86.7		90.6	
(range)	(63–269)		(42–205)		(45–195)	
**Weight lost (%)**						
Mean			32.9		25.3	
(range)			(−53.8 – +70.1)		(−27.5 – +68.5)	
**BMI (weight/height**^**2**^**)**						
Mean	41.3		30.4		31.9	
(range)	(24.9–79.2)		(15.8–65.4)		(17.3–65.9)	
**Smoking**						
Yes	3305	(11.1)	2343	(13.0)	489	(11.2)
Former smoker	5438	(18.2)	2723	(15.1)	663	(15.3)
No	19,391	(64.9)	9356	(52.0)	2328	(53.6)
Missing	1743	(5.8)	3108	(17.3)	381	(8.8)

*Gastric bypass (GBP).

** Sleeve gastrectomy (SG).

***Biliopancreatic diversion with duodenal switch (DS).

**Table 2:** Haemoglobin levels and percent with anaemia prior to surgery, and at follow-up at five and ten years, respectively.

	Prior to surgery	Follow-up 5 years	Follow-up 10 years
**Total population**			
Mean Hb (g/l)	140.7	131.8	131.6
(range)	(45–190)	(40–185)	(53–175)
Number	29,877	17,993	4347
% with anaemia*	4.1%	18.3%	18.9%
Patients with Hb from start, with Hb also at 5 and 10 years	29,877	9,392	474
Mean Hb (g/l)) based on the start group	140.7	132.2	131.6
Patients with Hb at 5 years, with Hb also at start and 10 years	9,392	17,993	2662
Mean Hb (g/l)) based on the 5 year group	140.6	131.8	131.5
Patients with Hb at 10 years, with Hb also at start and 5 years	474	2662	4347
Mean Hb (g/l)) based on the 10 year group	140.1	130.4	131.6
**Divided by sex**			
Women			
Mean Hb (g/l)	137.1	128.3	129.1
(range)	(75–181)	(50–183)	(53–172)
Number	22,943	13,944	3400
% with anaemia*	4.8%	20.3%	18.9%
Men			
Mean Hb (g/l)	152.5	143.8	140.5
(range)	(45–190)	(40–185)	(68–175)
Number	6934	4049	947
% with anaemia*	1.6%	11.2%	18.8%
Divided by sex and with anaemia*			
Women			
Mean Hb (g/l)	113.6	108.5	108.8
(range)	(75–119)	(50–119)	(53–119)
Number	1109	2838	644
Men			
Mean Hb (g/l)	122.4	119.4	117.8
(range)	(45–129)	(40–129)	(68–129)
Number	113	456	178
**Divided by method of surgery**			
GBY**			
Mean Hb (g/l)	140.9	131.4	131.6
(range)	(81–189)	(40–184)	(53–175)
Number	23,453	15,737	4271
SG***			
Mean Hb (g/l)	139.9	134.8	137
(range)	(45–190)	(81–185)	(115–159)
Number	6248	2053	12
DS****			
Mean Hb (g/l)	144	131.7	128.6
(range)	(115–179)	(97–168)	(58–165)
Number	176	203	64

*Anaemia defined as Hb < 120 g/L for women and Hb < 130 g/L for men.

**Gastric bypass (GBP).

*** Sleeve gastrectomy (SG).

****Biliopancreatic diversion with duodenal switch (DS).

In the mixed models analysis, we included the Hb level as the dependent variable and added fixed effects of time point for follow-up, gender, age, operation method and their interaction. We included each subject as a random effect. There was a significant effect of time point, gender, age and operation method and their interactions (*p* > 0.001 for all). The mean Hb levels were significantly different from each other; at the start (144.5 g/l), at the five- (136.4 g/l) and ten-year (135.2 g/l) follow-up (*p* < 0.001 for all comparisons, i.e., start vs 5 years, start vs ten years and five vs ten years, respectively. To compare the groups with and without Hb at inclusion a dropout analysis was also carried out comparing age at surgery, BMI at the three time points and Hb at five- and ten-years follow-up. See Table 3. As can be seen, patients that had their Hb registered had lower BMI and higher Hb but the difference decreased at ten years.

**Table 3:** Dropout analysis.

		*N*	Mean	SD	*P*-value
Hb at istart	No Hb	29,680	41.0	10.9	0.000
	Hb	29,877	41.0	11.2	
Hb at five years	No Hb	8601	131.3	15.0	0.001
follow-up	Hb	9392	132.2	14.4	
Hb at ten years	No Hb	3873	131.6	14.4	0.786
follow-up	Hb	474	131.6	14.5	
BMI at start	No Hb	29,680	42.8	5.8	0.000
	Hb	29,877	41.3	5.5	
BMI at five years	No Hb	15,878	30.4	5.4	0.010
follow-up	Hb	13,358	30.1	5.4	
BMI at ten years	No Hb	7162	31.7	5.9	0.025
follow-up	Hb	885	30.7	5.5	

The percentage with anaemia was 18.3 in the whole group after five years compared to 4.1% prior to surgery. The mean Hb levels in the total population of women at ten years follow-up were 129.1 g/L and for men 140.5 g/L, respectively. The mean Hb in the groups with anaemia was 108.5 and 108.8 g/L for women and 119.4 and 117.8 g/L for men after five and ten years, respectively. See Table 2.

For the total population with a registered ten-year follow-up, as well as for the men and women separately, more patients used supplementary treatment with iron after ten years than after five years. The use of oral iron went from 40 to 45% at follow-up at ten years and the need for parenteral iron intake went from 5 to 11% at follow-up at ten years. See Table 4.

**Table 4:** Supplementary treatment reported in the populations with five- and ten-years follow-up.

	Follow-up 5 years	Valid percent	Follow-up 10 years	Valid percent
Multivitamin*				
Yes	18,007	87	5048	78
No	2727	13	1379	22
Missing	8502		1620	
**Oral iron**				
Yes	7653	40	2923	45
No	11,686	60	3251	55
Missing	9897		1873	
**Parenteral iron**				
Yes	868	5	611	11
No	16,565	95	4993	89
Missing	11,803		1873	
Complete multivitamin**				
Yes	576	14	193	13
No	3638	86	1317	87
Missing	25,022		6537	
**B12**				
Yes	19,678	93	6042	91
No	1540	7	568	9
Missing	8018		1437	
**Folic acid**				
Yes	4146	22	1708	29
No	14,444	78	4259	71
Missing	10,646		2080	
**Vitamin D and calcium**				
Yes	16,233	78	5034	78
No	4577	22	1418	22
Missing	8426		1595	

*With or without iron.

**designed to contain complete substitution in one pill after bariatric surgery.

In both women and men, more patients needed substitution with iron as time went by. In both women and men, the substitution with oral iron differed, 44 compared to 50% in women and 25 to 37% in men after five and ten years, respectively. The corresponding numbers for parenteral iron were 6 compared to 12% in women and 2 to compared 5% in men, respectively. See Table S1A, B. Furthermore, substitution with multivitamins and vitamin D was also substantial as well as B12 for obvious reasons. See Table 4 and S1.

The prevalence of stomal ulceration was 1–2% in all groups. The use of PPI was 14% after ten years compared to 10% after five years. See Table S2. After ten years in women with anaemia 76 out of 644 patients (12%) were treated with PPI and in men 29 out of 178 (16%) were treated with PPI.

## Discussion

The purpose of bariatric surgery is to achieve weight loss and gain health benefits, but at the same time, the surgery increases the risk of nutritional deficiencies. One of the more potentially serious complications is anaemia. The percentage of anaemia increased from 4.1 before surgery to 18.9% after 10 years. Since the incidence of anaemia increases with age this could very well be caused by multimorbidity in an aging population. However, in a Danish study (based on cut-off levels 117 g/l for women and 134 g/l for men, respectively) the percentage with anaemia at 40–49 years of age was 8.1% and 3.9% in women and men, respectively compared to 4.4% and 6.8% in the age group 50–59, respectively [19]. Consequently, even though the Hb levels in the present study could be maintained to some degree, anaemia per se was apparent after a longer follow-up period. This observation is also in line with the findings in a Swedish SOS study from 2021 where anaemia was more frequent in those who had undergone bariatric surgery compared to ordinary non-surgical obesity care [17].

Even though the Hb levels drop to some extent in the present study, the proportion of patients with anaemia does not increase to any major extent over time compared to other studies based on patients who have been operated on with bariatric surgery. In an older study from 1998, 47% had anaemia and the Hb levels decreased from 138 to 124 g/l after ten years [15]. In 2014 another study found anaemia in 51% with a fall in Hb from 135 to 116 g/l [14], also after ten years. The Hb levels found in the present study were 141 g/l prior to surgery and 132 g/l after ten years (129 g/l in women and 140 g/l for men, respectively). Anaemia was more frequent in women (19%) compared to 16% in a recently published Norwegian study. The occurrence of anaemia in men cannot be compared since the definition of anaemia for men differs in the two populations [16]. Consequently, even though the preoperative level was slightly higher in the present study it seems as if the Hb level can be kept at a rather normal level despite the potential effects of bariatric surgery. This is probably due to an increased awareness among the healthcare professionals responsible for the follow-up visits. The information to patients about the importance of supplementary treatment with iron and other supplements after bariatric surgery also seems to be satisfactory.

However, to maintain homoeostasis a large minority must be prescribed different types of supplements. The need for substitution is not reduced as time goes by. In contrast, the group that is prescribed iron or folic acid increases over the years—even after ten years. However, this population is not regaining the blood level prior to surgery over time. This indicates that the prescribed doses of iron might be insufficient or that other causes than iron deficiency could be present. This suggests that a more accurate investigation of the cause of anaemia in this population in the first years after surgery should be undertaken and hopefully result in a higher Hb level than what has been achieved with the current guidelines.

Consequently, to prevent complications, a regular follow-up over a longer time span is mandatory. With a structured follow-up, individuals with any signs of malnutrition can be identified and referred for accurate treatment. The follow-up can also be a control station to make sure that the individuals have understood the importance of a lifelong substituting therapy post-bariatric surgery to reduce the risk of anaemia and other malabsorptive conditions in the future. It can be assumed that not all patients attend their follow-up visits. In view of the risk for malnutrition and anaemia in particular it is important to emphasise for the patient that regular visits are valuable to maintain health and quality of life.

The incidence of diagnosed stomal ulceration is low and even if it is underdiagnosed it is unlikely to contribute to the development of anaemia to any major degree. The use of PPI is high, especially among women after ten years where 13% were prescribed PPI. In view of the nature of the surgery that results in a vast reduction of acid production per se, this treatment does probably not play any major role in the development of anaemia. This is in line with the observation that the percentage of patients with PPI treatment did not differ between anaemic and non-anaemic patients. The indication for PPI treatment is not registered in SOReg but includes reflux disease and upper GI pain. The cause of anaemia after bariatric surgery is more likely to be found in altered anatomy and/or poor nutritional intake.

A strength of the present study is that almost all patients that have undergone bariatric surgery in the country have been included. As a result, the number of included patients is, to the best of our knowledge, the highest this far for a bariatric operated cohort. The long follow-up period covering a decade is also valuable and gives information about the risks over a longer time span. An important limitation of this study is that Hb has not been registered for all patients at all three-time points. This is due to the fact that Hb was introduced as a variable in SOReg 2012 and consequently no patients registered during the first five years have a preoperative Hb. The other factor of importance is that Hb is not a mandatory variable, and patients can be registered in SOReg without Hb. In a dropout analysis, patients who had their Hb registered seemed to have lower BMI and higher Hb, at least after five years, while the differences disappeared after ten years. In other words, there could be a risk that the results underestimate the magnitude of the anaemia. On the other hand, another possibility is that some patients did not come for follow-up because they felt all well, something that confers a risk that the number with anaemia instead could be too high (patients’ dropout), while the systematic lack of data from some centra results in an increased uncertainty (doctors’ dropout). Even though the number of dropouts was high with this design we still wanted to include all patients that had undergone bariatric surgery that had analysed Hb at any of the time points to illustrate the magnitude of the problem. Regardless of all these uncertainties the number of included Hb analyses is high and the fact that anaemia really is a problem as time goes by cannot be denied.

The severity of the anaemia can to some extent be illustrated by the need for intravenous treatment. However, since data has been retrieved from a registry any cases of hospitalisation are not possible to include in the analysis. Another limitation is that we only have information about the Hb and not the iron or ferritin levels. Our findings can hence not give any clear indication to all possible causes leading to anaemia. Another limitation is that the supplementary treatment with “multivitamins” can be both with or without iron. This group with multivitamin supplementation is thus stated separately from those with oral iron supplementation. However, multivitamins on the Swedish market contain only small amounts of iron, up to 15 mg/day in recommended doses. Furthermore, as in all registries, not all parameters are recorded and consequently data is missing. On the other hand, the large group may still provide information that makes the conclusions generalisable. Despite the high number of participants, where many patients have been registered on all three occasions, it is not a retrospectively collected prospective study but instead a cross-sectional study comparing three independent groups of patients. In view of the many participants, we consider it is still possible to draw conclusions about the occurrence of anaemia in patients that had undergone bariatric surgery.

To conclude, bariatric surgery is an effective measure to obtain weight loss and improved metabolic control. Despite regular follow-up visits and prescription of adequate substitution, anaemia is a significant but treatable problem and seems to be less pronounced compared to other reports. Regular check-up on a yearly basis is recommended to avoid serious complications such as untreated anaemia. However, even though supplements are prescribed to a substantial subgroup it has not been possible to fully restore the Hb levels to their original levels. Adjustments of the doses or in some cases a more thorough investigation of the underlying causes could be contemplated. In these patients, several causes behind anaemia could be present at the same time. In view of the large minority requiring substitution, it is recommended that the importance of regular follow-up visits is emphasised and that the medication is maintained over time. In view of the high incidence of anaemia after such a long time span as ten years, it is extra important to be aware of the substantial risk for the development of anaemia and malabsorption as time goes by. A patient that has gone through surgery for better health should not have to risk developing other diseases instead that impairs the quality of life.

## Conclusions

Anaemia is a significant but still manageable condition five and ten years after bariatric surgery. However, despite the prescription of oral iron supplements to 45% of the patients ten years after surgery, the Hb levels could still not be fully restored. Consequently, the importance of follow-up visits and continuous evaluation of the need for supplementation is emphasised.

## Supplementary information

S1

S2

## Data Availability

The data in this study has been retrieved from the SOReg registry (SOReg (uu.se)), the Scandinavian Obesity Surgery Registry.
